# Effect of the Functional Group Position in Functionalized Liquid Butadiene Rubbers Used as Processing Aids on the Properties of Silica-Filled Rubber Compounds

**DOI:** 10.3390/polym13162698

**Published:** 2021-08-12

**Authors:** Donghyuk Kim, Gyeongdong Yeom, Hongil Joo, Byungkyu Ahn, Hyun-Jong Paik, Heungbae Jeon, Wonho Kim

**Affiliations:** 1 School of Chemical Engineering, Pusan National University, Busan 46241, Korea; ehdgurzxc@gmail.com; 2 Department of Polymer Science and Engineering, Pusan National University, Busan 46241, Korea; rudehd2012@gmail.com; 3 Department of Chemistry, Kwangwoon University, Seoul 01897, Korea; jhi940307@naver.com; 4 Hankook Tire & Technology Co., Ltd. R&D Center, Yuseong-gu, Daejeon 34127, Korea; bkahn8855@gmail.com

**Keywords:** liquid butadiene rubber, silica-filled compound, anionic polymerization, radical polymerization, rubber compounding

## Abstract

Recently, research conducted on tread compounds with liquid butadiene rubber (LqBR) have been conducted in the tire industry. In particular, the introduction of functional groups into LqBRs is expected to lower hysteresis loss caused by the free chain ends of LqBR. To study this, LqBRs with functional groups at different positions were synthesized. The occurrences of in-chain and chain-end functionalization of functionalized LqBRs (F-LqBRs) were confirmed, the microstructure and functionalization efficiency of F-LqBRs were calculated through the characterizations. This novel functionalization technology was beneficial not only to immobilizing the free chain ends of LqBRs to the surfaces of silica to decrease the number of free chain ends, but also chemically bonding the LqBR chains on the base polymer through a crosslinking reaction to enhance the filler-rubber interaction. The effects of the functional group position and number of the free chain ends on the physical properties and hysteresis of the compounds were investigated by partially replacing the treated distillate aromatic extract (TDAE) oil with LqBR in silica-filled rubber compounds. The results showed that compounds that had applied DF-LqBR with both end functionalization performed better, including improving the silica dispersion, higher extraction resistance, and lower rolling resistance, than other F-LqBRs compounds.

## 1. Introduction

Liquid butadiene rubber (LqBR) is widely used as a vulcanizable plasticizer in tires, plastics, printing inks, paints, coatings, and sealants [1]. Interest in LqBR has increased significantly since 1990, as it has been used to serve the purpose of plasticizers and cure coagents, as well as to improve the viscoelastic properties of tire treads in the rubber industry. In particular, research in the tire industry has significantly increased over the past decade, which can be confirmed by the significant increase in the number of patent applications related to liquid polymers [2].

Since 1995, European processing oil producers and tire manufacturers have developed processing aids to replace highly aromatic oil (distilled aromatic extract, DAE), which contains carcinogenic polycyclic aromatic hydrocarbons (PAHs). In fact, the use of DAE oils in tire manufacturing was banned in 2010, according to the EU REACH regulations. Therefore, oils with low PAH content, such as treated DAE (TDAE) oils, are used as alternative processing aids [3,4]. However, TDAE oil-applied vulcanizates have a disadvantage in that the physical properties of vulcanizates deteriorate owing to the migration of TDAE oil to the tire surface over time [5,6]. To address these issues, new processing aids with low T_g_, non-PAHs, and no migration problem have been suggested. Accordingly, the demand and interest in LqBRs have gradually increased.

Along with various attempts to apply LqBR as a processing aid in the manufacture of passenger car tires, application technologies have been gradually developed. Kuraray Co., Ltd. in Japan (2017), Sumitomo Rubber Industries in Japan (2018), and Continental AG in Germany (2015) improved the abrasion resistance, snow traction, and viscoelastic performance at low temperatures by applying non-functionalized LqBR (NF-LqBR) with low vinyl content to winter tire compounds [7,8]. Hirata et al., applied commercialized non-functionalized liquid rubbers as processing aids to carbon black-filled NR compounds, thereby overcoming the processability and migration problems [9]. However, the problem of hysteresis caused by the free chain ends of LqBRs should be solved to expand the application of LqBRs in passenger car tires.

With interest in non-DAE plasticizers, tire manufacturers led by Michelin conducted studies using silica as a reinforcing filler to replace carbon black in order to reduce greenhouse gas emissions in 1993 [10]. Due to the fact that the silica surface is significantly more reactive than that of hydrophobic carbon black, the use of silane coupling agents was necessary for compounds to achieve a desirable balance of traction and low rolling resistance. Therefore, this switch in filler technology was responsible for increasing interest in the introduction of functional groups into polymer chains [11,12,13].

Based on these technologies, silane-functionalized LqBRs have been commercialized since 2017 [14,15], and studies on their application to tire compounds have been reported [16,17,18]. Salort et al., reported that low-molecular-weight silane-functionalized LqBRs can be crosslinked to the base polymer by sulfur through higher molecular-weight structure formation by self-condensation [2]. Kim et al., reported that silane-functionalized LqBRs can perform a role similar to that of a silane coupling agent in the compound because the ethoxysilyl group not only reacts with the silanol group of silica, but also crosslinks with the base polymer by consuming sulfur [19]. In addition, when processing oils and NF-LqBRs are replaced by silane-functionalized LqBRs, hysteresis can be greatly reduced owing to the decrease in the chain mobility due to the functional groups and the decrease in the number of free chain ends. Moreover, Hogan et al., reported that additional modifications of LqBR with alkoxy groups, amino groups, cyano groups, sulfonyl groups, epoxy groups, and halogen atoms further improved performance, as it can form chemical bonds with silanol groups present on the silica surface [11,12,13].

However, most previous studies used commercialized functionalized-LqBRs (F-LqBRs) instead of in-house synthesized LqBRs, so the structure of LqBRs was limited with respect to the control of molecular weight, vinyl content, and functionalization. Due to this limitation, it was difficult to explain the underlying reason for the mechanism of F-LqBRs in the compounds and physical properties. It is a technical problem in the synthesis of F-LqBRs that needs to be investigated. The synthesis technique of the introduction of functional groups into LqBRs used in this article is very rare. There are few related studies on this. In addition, there is still a lack of research on the effect of the functional group position and number of the free chain ends of F-LqBRs on the physical properties and hysteresis of the compounds. Moreover, many patents describe the performances of the tire tread compound that include F-LqBRs. However, there are no publications that describe the reasons for the observed performances. A better understanding of it would lead to more effective developments on the structure of F-LqBRs and applications in tire tread compounds. Therefore, the goal of the present investigation is to provide more understanding about the effects of F-LqBRs in tread compounds: (i) influence of the functional group position in F-LqBRs on silica hydrophobation and physical properties; (ii) the effect of the number of free chain ends of F-LqBRs on the hysteresis of the compounds.

To address these problems, in this study, LqBRs with low vinyl content and functional groups at different positions, such as NF-LqBR, one-chain end-functionalized LqBR (OF-LqBR), center-functionalized LqBR (CF-LqBR), and di-functionalized LqBR (DF-LqBR), were synthesized using anionic and radical polymerization. In addition, silica-filled rubber compounds were manufactured by applying LqBRs as processing aids to replace TDAE oils, and the effects of the functional group position and number of free chain ends of LqBRs on the performance of tire tread compounds were confirmed by evaluating the physical properties. Moreover, the mechanism and physics of the structure formation and the relationship with the properties were analyzed in detail. The presented results are expected to provide a foundation for the optimal design of LqBR structure and selection of LqBRs required for the manufacture of tire tread compounds to which LqBRs are applied.

## 2. Materials and Methods

### 2.1. Materials

#### 2.1.1. Synthesis of 4,4′-(diazene-1,2-diyl)bis-(4-cyano-N-(3-triethoxysilyl)propyl)pentanamide (Difunctional Initiator)

Triethylamine (TEA, 98%, Duksan General Chemical Co., Seoul, Korea), 4,4′-azobis(4-cyanovaleric acid) (98%, Sigma-Aldrich Corp., Seoul, Korea), (3-aminopropyl) triethoxysilane (98%, Sigma-Aldrich Corp., Seoul, Korea), and ethyl chloroformate (97%, Sigma-Aldrich Corp., Seoul, Korea) were used as reagents. Tetrahydrofuran (THF, 99%, Duksan General Chemical Co., Seoul, Korea) was used as a solvent. For purification, *n*-hexane (95%, Duksan General Chemical Co., Seoul, Korea) and diethyl ether (99%, Daejung Chemicals & Metals Co., Siheung, Korea) were used.

#### 2.1.2. Polymerization

All materials used in the polymerization were purged with nitrogen, and cyclohexane (99%, Samchun Chemical Co., Seoul, Korea) and tetrahydrofuran (THF, 99%, Samchun Chemical Co., Seoul, Korea) were used as organic solvents. *N*-butyl lithium (2.0 M in cyclohexane, Sigma-Aldrich Corp., Seoul, Korea) and a difunctional initiator (self-made in laboratory) were used as initiators for anionic and radical polymerization. Anisole (99%, Samchun Chemical Co., Seoul, Korea) was used as a polar modifier to control the vinyl content, and 1,3-butadiene (Kumho Petrochemical Co., Daejeon, Korea) was used as a monomer. Tetraethyl orthosilicate (TEOS, 98%, Sigma-Aldrich Corp., Seoul, Korea) and 3-chloropropyltriethoxysilane (CPTES, 97%, Sigma-Aldrich Corp., Seoul, Korea) were used as coupling agents, and *n*-octyl alcohol (99%, Yakuri Pure Chemicals Co. Ltd., Kyoto, Japan) was used as a terminating agent.

#### 2.1.3. Compounding

SSBR (SOL-5220M, Kumho Petrochemical Co. Daejeon, Korea, styrene content: 26.5 wt%, vinyl content: 26 wt%, non-oil extended) and high-cis butadiene rubber (CB24, Lanxess Chemical Industry Co., Ltd., Cologne, Germany; cis content: 96 wt%) were used as base polymers. Silica (ZEOSIL 195MP, Solvay Silica Korea Co., Ltd., Gunsan, Korea) was used as a filler, and X50-S (Evonik Industries AG, Essen, Germany; bis-[3-(triethoxysilyl)propyl]tetrasulfide (TESPT) 50%, carbon black N330 50%) was used as a silane coupling agent. Moreover, TDAE oil (Kukdong Oil & Chemicals Co., Yangsan, Korea) was used as a processing aid for mixing. ZnO and stearic acid (both from Sigma-Aldrich Corp., Seoul, Korea) were used as activators, and N-(1,3-dimethylbutyl)-N-phenyl-p-phenylenediamine (6PPD, Kumho Petrochemical Co., Daejeon, Korea) was used as an antioxidant in the compound. Sulfur (Daejung Chemicals & Metals Co., Siheung, Korea) was used as a crosslinking agent. N-cyclohexyl benzothiazole-2-sulfenamide (CBS, 98%, Tokyo Chemical Industry Co. Ltd., Tokyo, Japan) and 1,3-diphenylguanidine (DPG 98%, Tokyo Chemical Industry Co. Ltd., Tokyo, Japan) were used as cure accelerators.

### 2.2. Measurements

#### 2.2.1. Gel Permeation Chromatography (GPC)

The molecular weight and the molecular weight distribution were measured using a gel permeation chromatography (GPC) system (Shimadzu, Kyoto, Japan). The GPC system consisted of a solvent delivery unit, a refractive index detector, and three types of Styragel columns: HT 6E (10 µm, 7.8 mm × 300 mm), HMW 7 column (15–20 μm, 7.8 mm × 300 mm), and HMW 6E column (15–20 μm, 7.8 mm × 300 mm). The measured molecular weight was corrected using a polystyrene standard sample (Easi Cal PS-1 standard, Agilent Technologies, Santa Clara, CA, USA).

#### 2.2.2. Proton Nuclear Magnetic Resonance Spectroscopy (^1^H NMR)

The vinyl content in LqBR was evaluated using proton nuclear magnetic resonance spectroscopy (^1^H NMR; Varian, Unity Plus 300 spectrometer, Garden State Scientific, Morristown, NJ, USA). LqBRs were dissolved in a 5 mm NMR tube at a concentration of 15 mg/mL using deuterochloroform (CDCl_3_, Cambridge Isotope Laboratories, Inc., Andover, MA, USA) as a solvent.

#### 2.2.3. Differential Scanning Calorimetry (DSC)

The glass transition temperature (T_g_) was determined using a differential scanning calorimeter (DSC-Q10, TA Instruments, New Castle, DE, USA). The curves for samples (3–6 mg) were obtained by heating the sample from −120 to −20 °C at a heating rate of 10 °C/min under a nitrogen atmosphere.

#### 2.2.4. Payne Effect

A rubber processing analyzer (RPA2000, Alpha Technologies, Hudson, OH, USA) was used to evaluate the silica dispersion and filler–filler interaction of the compound. The storage modulus (G′) of the uncured compound was measured at a temperature of 60 °C in the range of 0.01–40.04% strain. In the low-strain region, the G′ value is large because the silica agglomerates are not destroyed. In contrast, with the agglomerate destruction in the high-strain region, the G′ value decreases. Therefore, the change in G′, that is, ΔG′ (G′ at 0.28% − G′ at 40.04%) is called the Payne effect, which indicates the degree of the filler–filler interaction.

#### 2.2.5. Mooney Viscosity

A Mooney viscometer (Vluchem IND Co., Seoul, Korea) was used to evaluate the processability of rubber by measuring the torque when the rotor rotated in a space filled with an uncured rubber compound according to ASTM D1646. The rotation speed of the rotor was set to 2 rpm, and the temperature was set to 100 °C. The torque value was measured by rotating the rotor for 4 min after preheating for 1 min.

#### 2.2.6. Cure Characteristics

To measure the curing characteristics of the compound, a moving die rheometer (MDR, Myung Ji Co., Seoul, Korea) was operated for 30 min, maintaining a vibration angle of ±1° and a temperature of 160 °C. Meanwhile, the minimum torque (T_min_), maximum torque (T_max_), scorch time (t_10_), and optimal cure time (t_90_) were measured.

#### 2.2.7. Solvent Extraction and Crosslink Density

The crosslink density is defined as the number of crosslink points in vulcanizates. If the crosslink density is increased, the molecular weight between crosslink points decreases, and the number of crosslink points increases. In swelling tests, the higher the crosslink density, the lower the number of solvent molecules that can permeate between the crosslinked rubber chains, resulting in relatively less swelling. Vulcanizates with dimensions of 10 mm × 10 mm × 2 mm were weighed and immersed in tetrahydrofuran (THF, 99%, Samchun Chemical Co., Seoul, Korea) and *n*-hexane (95%, Samchun Chemical Co., Seoul, Korea) at 25 °C for 2 days to remove organic additives from the sample. Subsequently, the weight was measured after the sample was dried at 25 °C for 1 day to determine the mass fraction of the extracted organic additive. The weight of the sample immersed and swollen in a toluene solvent for 1 day at room temperature (25 °C) was measured after measuring the weight of the dry sample to calculate the total crosslink density. Moreover, the total crosslink density and the average molecular weight between crosslink points, M_c_, were calculated using the Flory–Rehner equation [20,21,22,23]:

(1) $$\nu =\frac{1}{2M_{c}}=-\frac{\ln\left(1-v_{r}\right)+v_{r}+\chi v_{r}^{2}}{2\rho V_{s}\left(v_{r}^{\frac{1}{3}}-v_{r}/2\right)},$$

where *ν* is the crosslink density (mol/g), *M_c_* is the average molecular weight between crosslink points (g/mol), *ν_r_* is the volume fraction of rubber in the swollen gel at equilibrium given by Equation (2), vs. is the molar volume of the solvent (cm^3^/mol), *ρ* is the density of the rubber sample (g/cm^3^), and *χ* is the polymer–solvent interaction parameter.

(2) $$v_{r}=\frac{\frac{w_{dry}-w_{filler}}{\rho_{rubber}}}{\begin{array}{c} \frac{w_{dry}-w_{filler}}{\rho_{rubber}}+\frac{w_{swollen}-w_{dry}}{\rho_{solvent}} \end{array}}$$

In this equation, *w_dry_* is the weight of the dry sample, *w_filler_* is the weight of the filler in the dry sample, *w_swollen_* is the weight of the swollen sample, *ρ_rubber_* is the density of the rubber, and *ρ_solvent_* is the density of the solvent.

(3) $$\chi =0.34+\frac{v_{0}}{\begin{array}{c} RT \end{array}}{\left(\delta_{p}-\delta_{s}\right)}^{2}.$$

Here, *ν*_0_ is the molar volume of the solvent, *δ_p_* is the solubility parameter of the polymer, and *δ_s_* is the solubility parameter of the solvent.

#### 2.2.8. Mechanical Properties

A 100 mm (length) × 25 mm (width) dumbbell-shaped specimen that was prepared according to ATSM D 412 was measured at a speed of 500 mm/min using a universal testing machine (UTM, KSU-05M-C, KSU Co., Ansan, Korea) to measure the mechanical properties (tensile strength, modulus, and elongation at break) of the vulcanizates.

#### 2.2.9. Abrasion Resistance

The abrasion resistance was measured according to DIN 53516 using a Deutsche Industrie Normen (DIN) abrasion tester. The specimen was prepared in a cylindrical shape with a diameter of 16 mm and a thickness of 8 mm. The mass reduction after moving the specimen 40 m across the surface of an abrasive sheet mounted on a cylindrical drum revolving at 40 ± 1 rpm was measured by applying a load of 5 N.

#### 2.2.10. Viscoelastic Properties

For the dynamic viscoelastic properties of the compound, the storage modulus (G′), loss modulus (G″), and tan δ were measured from −60 to 70 °C under a 10 Hz frequency at 0.5% strain using a strain-controlled rheometer (ARES-G2, TA Instrument, New Castle, DE, USA).

### 2.3. Synthesis of 4,4′-(diazene-1,2-diyl)bis-(4-cyano-N-(3-triethoxysilyl)propyl)pentanamide (Difunctional Initiator)

**Scheme 1:**
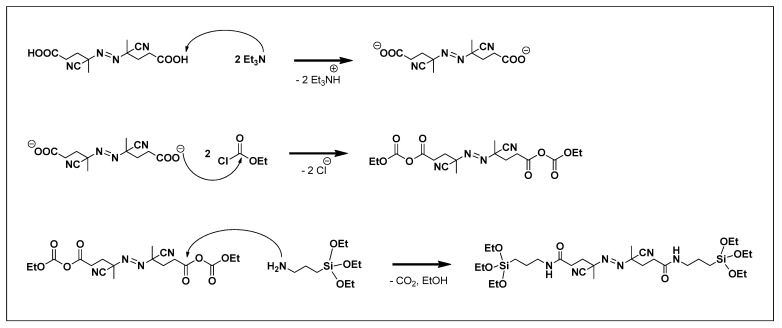
Synthesis of the difunctional initiator.

The synthesis of a difunctional initiator containing ethoxysilyl groups at both ends is shown in Scheme 1 [24]. 14 g (0.049 mol, 1 equivalent) of 4,4-azobis(4-cyanovaleric acid) was dissolved in 300 mL of THF at room temperature (25 °C) under Ar condition. After stirring at −78 °C for 20 min, 9.54 mL of ethyl chloroformate (0.099 mol, 2 equivalents) and 13.9 mL of triethylamine (0.099 mol, 2 equivalents) were added and stirred at −78 °C for 1 h. After adding 23.25 g of (3-aminopropyl)triethoxysilane (0.099 mol, 2 equivalents), the reaction mixture was stirred at 0 °C for 24 h. At the end of that period, the reaction mixture was filtered at room temperature. After the filtrate was concentrated under reduced pressure, hexane was added. The precipitate was collected and recrystallized from THF/hexane. The precipitates were washed with diethyl ether and dried under vacuum to obtain a pure product (difunctional initiator).

### 2.4. Synthesis and Functionalization of Liquid Butadiene Rubbers

#### 2.4.1. Anionic Polymerization

**Scheme 2:**
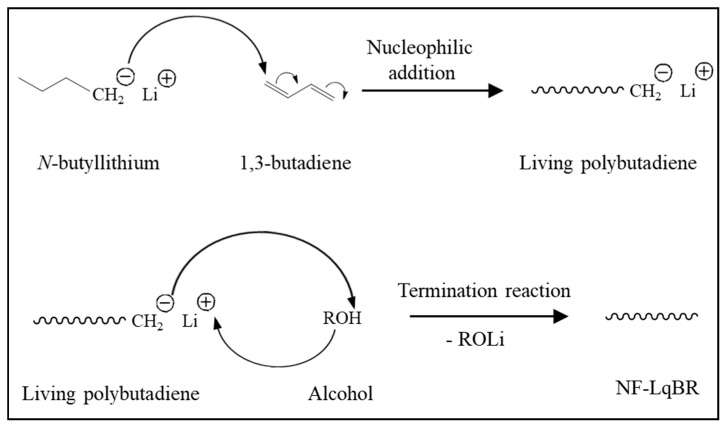
Polymerization of non-functionalized liquid butadiene rubber (NF-LqBR).

**Scheme 3:**
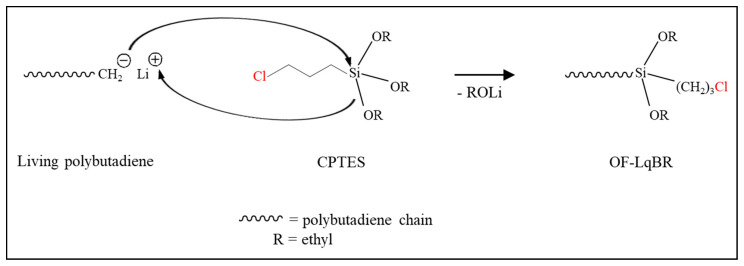
Polymerization of one-chain end-functionalized liquid butadiene rubber (OF-LqBR).

**Scheme 4:**
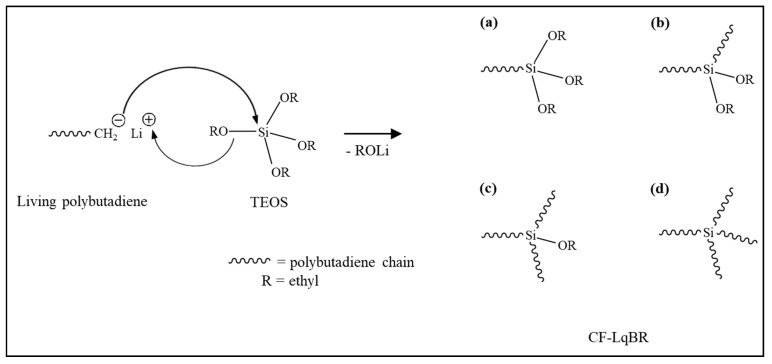
Polymerization of center-functionalized liquid butadiene rubber (CF-LqBR); (**a**) BRSi(OR)_3_, (**b**) BR_2_Si(OR)_2_, (**c**) BR_3_Si(OR), and (**d**) BR_4_Si.

Non-functionalized liquid BR (NF-LqBR), one-chain end-functionalized liquid BR (OF-LqBR), or center-functionalized liquid BR (CF-LqBR) were synthesized by anionic polymerization at 50 °C in a stainless-steel reactor (2 L) purged with nitrogen. The polymerization formulations are shown in Table 1. The amount of *n*-butyllithium was adjusted to synthesize them with similar molecular weights, and a 6 molar ratio of anisole to *n*-butyllithium was introduced to maintain the a low vinyl content [21]. Subsequently, 1,3-butadiene was introduced into the reactor under nitrogen pressure. Polymerization of all LqBRs, except for DF-LqBR, was carried out under the same reaction conditions. After 40 min, the reaction was terminated using *n*-octyl alcohol (1.2 molar excess to the initiator) (Scheme 2). The polymerization of OF-LqBR was terminated by adding CPTES to synthesize a structure in which the terminal ends of the BR chain were modified (Scheme 3) [25,26,27,28]. In contrast, the polymerization of CF-LqBR was terminated by adding tetraethyl orthosilicate (TEOS, 0.5 molar ratio) to synthesize a structure in which two BR chains were coupled. (Scheme 4) [22,25]. Finally, LqBRs were obtained by evaporating cyclohexane in LqBR solutions using a vacuum evaporator.

#### 2.4.2. Radical Polymerization

The polymerization of 1,3-butadiene was performed in a high-pressure stainless-steel reactor (1 L) with a stirrer. First, the reactor was charged with 0.529 g of silane azo initiator and 416 g of THF, and purged with nitrogen gas. Next, 83.3 g 1,3-butadiene (measured in a small chamber) was injected into the reactor under a nitrogen pressure of 4 bar. The polymerization was carried out for 48 h at a temperature of 70 °C. After the reaction, the reaction mixture was cooled, and the remaining butadiene was vented from the reactor. THF was removed using rotary evaporation to concentrate the polymer solution. To remove the residual initiator, the solution was poured into ethanol to precipitate the polymers. Finally, the polymers were collected by centrifugation. 

**Scheme 5:**
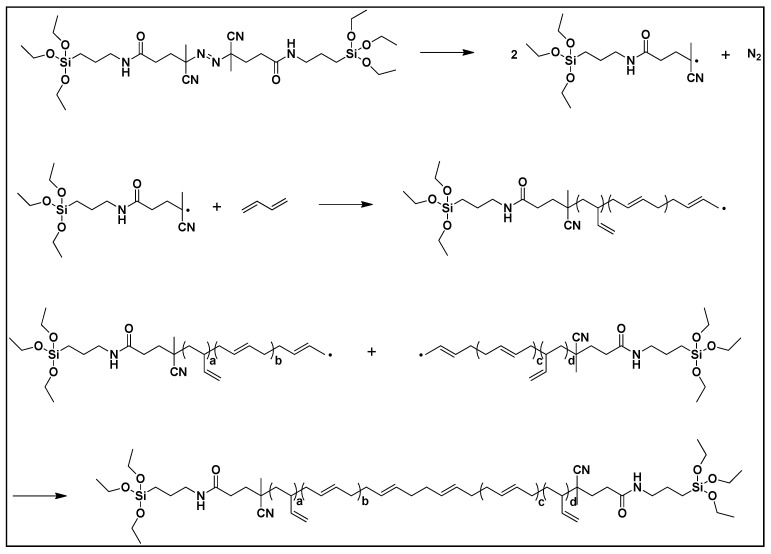
Polymerization of di-functionalized liquid butadiene rubber (DF-LqBR).

The propagating polymer chains can terminate in three ways: coupling, disproportionation, and chain transfer. If there is no chain transfer agent, the polymer chains terminate by coupling and disproportionation. In the case of butadiene, it has been reported that termination occurs by coupling [29,30]. Therefore, we synthesized telechelic polybutadiene with triethoxysilane terminal groups using a free radical azo initiator containing triethoxysilyl groups (Scheme 5).

The macrostructure and microstructure of all LqBRs were analyzed by GPC and ^1^H NMR.

### 2.5. Preparation of Rubber/Silica Compounds and Vulcanizates

The compound was prepared using an internal mixer (300 cc, Mirae Scientific Instruments Inc., Gwangju, Korea) based on the formulations listed in Table 2. The fill factor was set to 70% of the mixer capacity. The input unit was parts per hundred rubber (phr), and the formulations were added based on the rubber content. A total of 10 phr of TDAE oil was replaced by adding LqBRs in all cases. The mixing procedures are shown in Table 3. The initial temperatures of each stage were 100 °C and 50 °C, respectively, and the dump temperatures were controlled at 150–155 °C and 80–90 °C, respectively. After mixing at each stage, the compound was sheeted using a two-roll mill. After confirming the optimal vulcanization time at 160 °C using an MDR, vulcanizates were prepared by pressing the prepared compounds in a hydraulic press at 160 °C for the optimal vulcanization time.

## 3. Results and Discussion

### 3.1. Synthesis of the Difunctional Initiator

**Figure 1:**
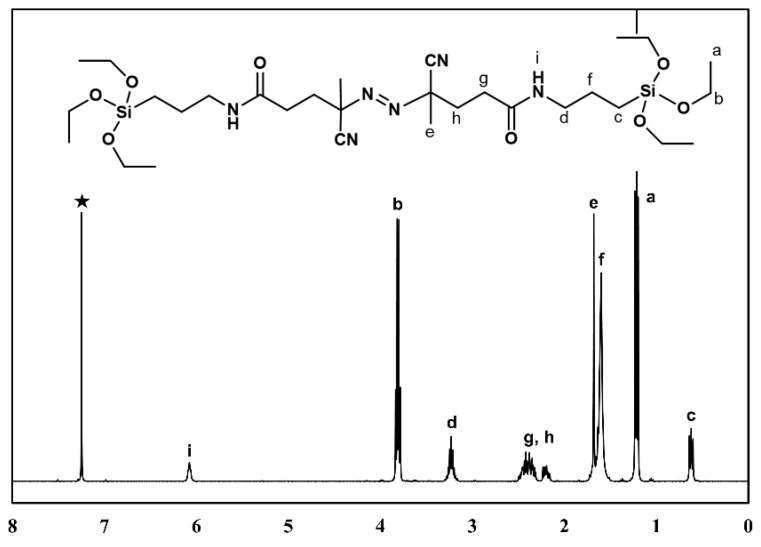
^1^H NMR spectra of the difunctional initiator (★ CDCl_3_ solvent).

Figure 1 shows the results of ^1^H NMR analysis of the difunctional initiator. In the difunctional initiator structure, the silyl ethoxy group 1.23 (t, 18H, SiO-CH_2_-C**H_3_**), 3.84 (q, 12H, SiO-C**H_2_**-), the methylene groups 3.52 (m, 4H, NH-C**H_2_**) next to nitrogen of amide group, 0.64 (t, 4H, Si-C**H_2_**-), 1.62 (q, 4H, Si-CH_2_-C**H_2_**) in silyl group, methylene groups group 2.16–2.52 (m, 8H, CO-CH_2_-C**H_2_**, CO-C**H_2_**), methyl peak of quaternary carbon 1.70 (s, 6H, C-C**H_3_**), and amide group 6.10 (s, 1H, N**H**) showed those ^1^H NMR resonance peaks.

Chemical shifts (δ) are reported with an internal standard, tetramethylsilane, and the the ^1^H NMR peaks are shown below: ^1^H NMR (CDCl_3_): δ ppm 0.64 (t, 4H, Si-C**H_2_**-), 1.23 (t, 18H, SiO-CH_2_-C**H_3_**), 1.62 (q, 4H, Si-CH_2_-C**H_2_**), 1.70 (s, 6H, C-C**H_3_**), 2.16–2.52 (m, 8H, CO-CH_2_-C**H_2_**, CO-C**H_2_**), 3.52 (m, 4H, NH-C**H_2_**), 3.84 (q, 12H, SiO-C**H_2_**-).

### 3.2. Synthesis of LqBRs

**Figure 2:**
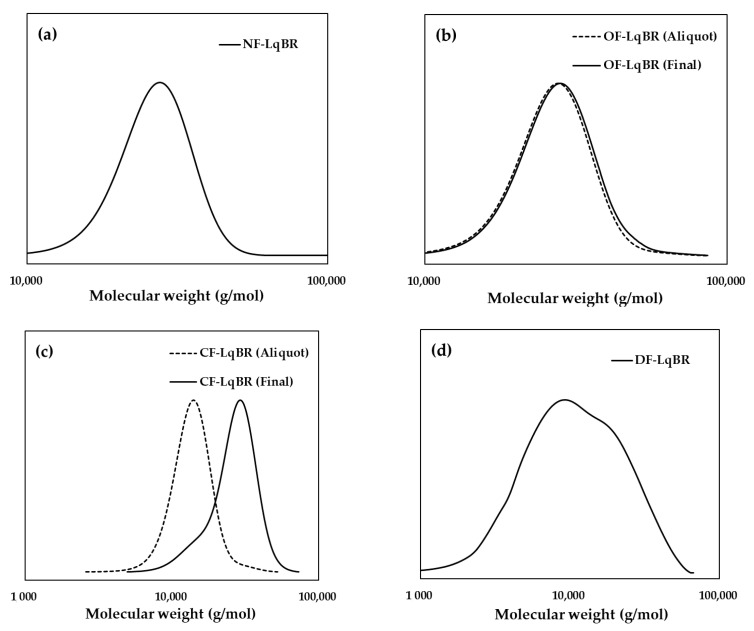
Gel permeation chromatography (GPC); (**a**) NF-LqBR, (**b**) OF-LqBR, (**c**) CF-LqBR, and (**d**) DF-LqBR.

**Figure 3:**
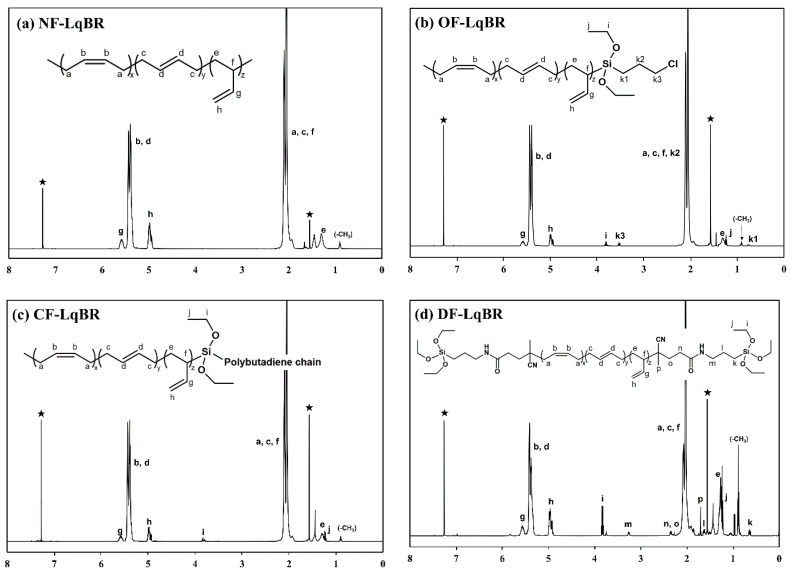
^1^H NMR spectra; (**a**) NF-LqBR, (**b**) OF-LqBR, (**c**) CF-LqBR, and (**d**) DF-LqBR ★ Resonance peak of CDCl_3_ at 1.57 and 7.26 ppm; due to solvent impurities and residual H.

Figure 2 and Figure 3, and Table 4 show the results of GPC and ^1^H NMR of the polymerized LqBRs. Kim et al., reported that most LqBRs can be crosslinked to the base polymer by sulfur when the molecular weight of the LqBR is 25,000 g/mol or higher [19]. In addition, Salort et al., reported that low-molecular-weight di-functionalized LqBRs can be crosslinked to the base polymer by sulfur through higher molecular-weight structure formation by self-condensation [2]. Both sets of study results clearly show that co-vulcanization with the base polymer is an important factor in determining the molecular weight of LqBRs. Based on the GPC results, NF-LqBR, OF-LqBR, and CF-LqBR synthesized by anionic polymerization had molecular weights ranging from 24,700 to 26,600 g/mol and a narrow molecular weight distribution (1.08–1.14). DF-LqBR synthesized by radical polymerization had a molecular weight of 7400 g/mol and a relatively broad molecular weight distribution (1.84). In the NMR spectra, resonance peaks appear at 5.37–5.50 ppm of the structure by 1,4-addition and 5.50–5.60 and 4.79–4.99 ppm of the structure (vinyl group) by 1,2-addition. The vinyl contents (anionic polymerization; 8–10 wt%, radical polymerization; 19 wt%) of LqBRs were calculated using their integration ratios [25]. ^1^H chemical shifts of ethoxy groups on the alkoxysilyl group appear at 1.19–1.26 ppm as a triplet (SiO-CH_2_-C**H_3_**) and at 3.75–3.85 ppm as a quartet (SiO-C**H_2_**-) [31]. The chloropropyl group of OF-LqBR showed peaks at 0.73–0.78 ppm (-Si-C**H_2_**-), 2.1 ppm (-Si-CH_2_-C**H_2_**-), and 3.48–3.54 ppm (-C**H_2_**-Cl), respectively [22,25]. The coupling number (CN), which represents the number of coupled polybutadiene chains of CF-LqBR, was calculated as the ratio of the number-average molecular weight (M_n_) before and after the coupling reaction [32]:

(4) $$\mathrm{Coupling}\;\mathrm{number}\;(\mathrm{CN})=\frac{\mathrm{Number}\;\mathrm{average}\;\mathrm{molecular}\;\mathrm{weight}\;\mathrm{after}\;\mathrm{coupling}}{\mathrm{Number}\;\mathrm{average}\;\mathrm{molecular}\;\mathrm{weight}\;\mathrm{before}\;\mathrm{coupling}}$$

The CN of CF-LqBR was 1.9, which indicates that the coupling reaction of the two polybutadiene chains (Scheme 4b) was dominant.

In the ^1^H NMR spectra, the area of the resonance peak for each H atom is proportional to the number of H atoms. According to this principle, the functionality, which is the ratio of the chain that contains the ethoxy-silyl group to the entire LqBR chain, was calculated as the integration of protons on the vinyl groups to the integration of protons on the alkoxylsilyl groups [33]:

(5) $$\frac{S_{Vinyl-H}}{S_{Alkoxysilane-H}}=\frac{2\times \left(R_{Vinyl}\right)\times \left(M_{n}/M_{B}\right)}{n_{Alkoxysilane}\times F}$$

where *S_vinyl-H_* and *S_Alkoxysilane-H_* are the peak areas of H atoms of vinyl and alkoxysilane, respectively; *R_Vinyl_* is the vinyl content; *M_n_* is the number-average molecular weight of LqBR; *M_B_* is the molecular weight of butadiene, *n_alkoxysilane_* is the number of H atoms of alkoxysilane, i.e., -Si-(OC**H_2_**CH_3_)_3_; “4”, “4”, and “6” are, respectively, for OF-LqBR, CF-LqBR, and DF-LqBR; and *F* is the functionality (Si/chain). For example, “2” indicates the two functionalized ends of macromolecular chains.

### 3.3. Manufacture of Rubber/Silica Compounds

**Figure 4:**
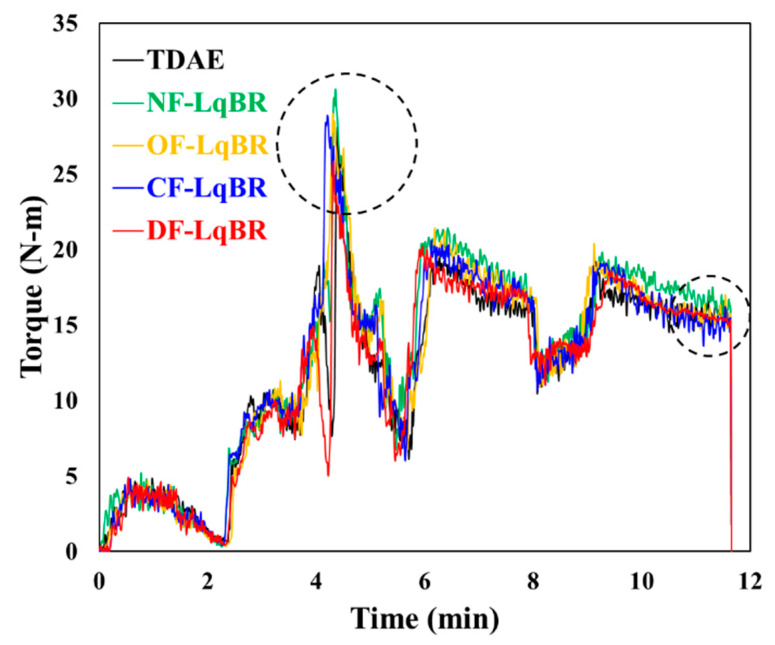
Mixing torque of the compounds during the first stage.

The compounds were mixed in two stages. The mixing torque of the compounds during the first step is shown in Figure 4 and Table 5. The mixing procedure of the first mixing stage includes the following steps (in min:s): 1. at 0:00 rubber addition; 2. at 1:20 addition of 50% of the silica, X50S, DPG, and TDAE or LqBR; 3. at 3:20 addition of the remaining 50% of the silica, X50S, DPG, and TDAE or LqBR; 4. at 5:20 addition of the zinc oxide, stearic acid, and 6PPD; 5. discharge at 11:40. The starting temperature of the mixer was set at a temperature of 100 °C. The targeted temperature at the end of the mixing was 150–155 °C for the compound to reach a silanization with the silane coupling agent. The second mixing step was carried out in the same mixer. The amounts of accelerators and sulfur were recalculated according to the mass of the compound after the first mixing stage.

### 3.4. Payne Effect

**Figure 5:**
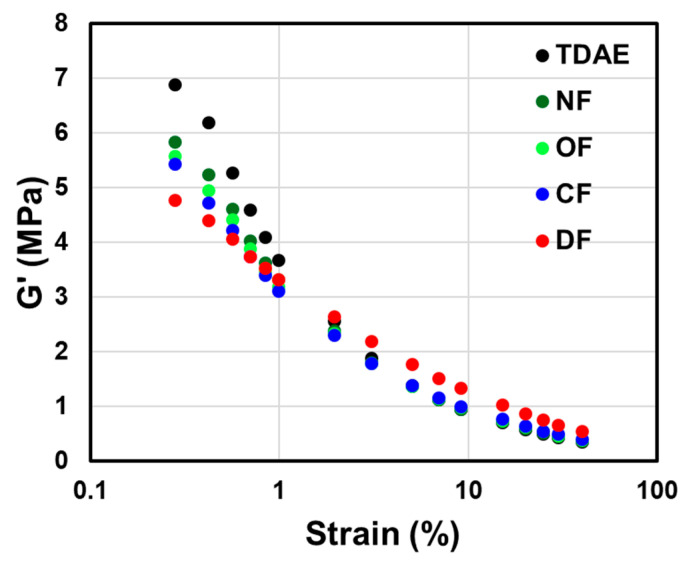
Storage modulus (G′) of the uncured compounds according to the applied processing aids.

The Payne effect presented in Figure 5 and Table 6 indicates the filler–filler interaction of the uncured compound. A decrease in the storage modulus (G′) with increasing strain amplitude is a result of the destruction of the filler network. The larger the ΔG′ value, the stronger the filler–filler interaction [34].

Sufficient viscosity and shearing force are required during the mixing process to achieve excellent silica dispersion within the compound [1,19]. Due to the fact that LqBRs, which have higher molecular weights than TDAE oils (molecular weight < 500 g/mol), possess higher viscosity, high peak torque values were obtained in the first stage of the mixing process, as shown in Figure 4 and Table 5. As a result, all compounds to which the LqBR was applied showed improved silica dispersion and a lower Payne effect, as compared to the compound with TDAE oils. In particular, OF-LqBR and CF-LqBR showed lower ΔG′ (0.28–40.04%, MPa) values than NF-LqBR, which has a similar molecular weight because of the improved dispersion, as the ethoxy group modifies the silica surface. In addition, DF-LqBR exhibited the lowest ΔG′ value owing to its excellent hydrophobation effect for silica surface.

### 3.5. Cure Characteristics and Mooney Viscosity

**Figure 6:**
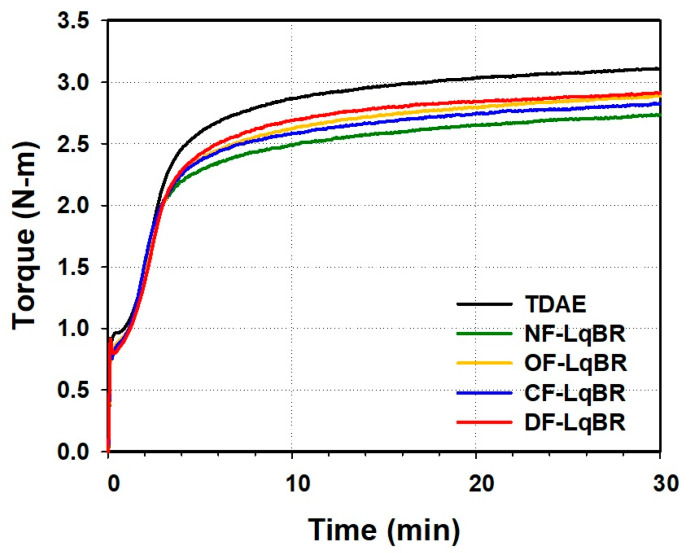
Cure curves of the compounds according to the applied processing aids.

The mixing process shown in Figure 4 is a method to maintain the dump temperature (150–155 °C) of the compound by adjusting the rotor RPM. Hence, it is not possible to compare the processability of each compound with the final torque value. Therefore, the Mooney viscosity, which is related to the processability of the compound, was measured. The Mooney viscosity results, and the cure characteristics obtained using an MDR are shown in Table 7 and Figure 6. LqBRs not only improve silica dispersion, but also show lower Mooney viscosity and minimum torque (T_min_) values, as compared with the TDAE compound, because chain slippage is facilitated by acting as a lubricant between the base polymer chains [1,19]. The compounds with OF-LqBR and CF-LqBR exhibited lower Mooney viscosity and T_min_ values than NF-LqBR owing to the improved silica dispersion. In particular, because the branched CF-LqBR has a smaller hydrodynamic radius than the linear OF-LqBR, it has a lower viscosity [35], and the Mooney viscosity and T_min_ values of the compound tend to decrease. In contrast, DF-LqBR, which has a low entanglement effect owing to its relatively low molecular weight, was expected to have the lowest viscosity [36,37,38], but rather showed similar values to that of TDAE compounds. This is because the amide and cyano groups at the chain end of DF-LqBR form strong hydrogen bonds with the amide and cyano groups of other DF-LqBR chains, which limits chain mobility and thereby increases viscosity [30].

In the case of silica compounds, the delta torque (ΔT, T_max_–T_min_) value in the MDR curve is affected by the filler morphology and the crosslink density [39]. In general, the ΔT values of NF-LqBR compounds are lower than those of TDAE oil because NF-LqBR consumes sulfur, which is used for the crosslinking reaction between the base polymers. In contrast, functionalized-LqBRs can increase filler–rubber interactions by not only fixing functional groups on the silica surface, but also forming chemical bonds with the base polymers through a crosslinking reaction with the base polymer. Furthermore, DF-LqBR forms a network structure by self-condensation between ethoxy groups, which limits chain mobility [5]. For this reason, it is considered that the compounds to which functionalized-LqBRs were applied showed higher ΔT values than NF-LqBR compounds, even though the silica dispersion was excellent. Therefore, their crosslink density values are expected to be higher than those of NF-LqBR compounds [40].

### 3.6. Solvent Extraction and Crosslink Density

**Figure 7:**
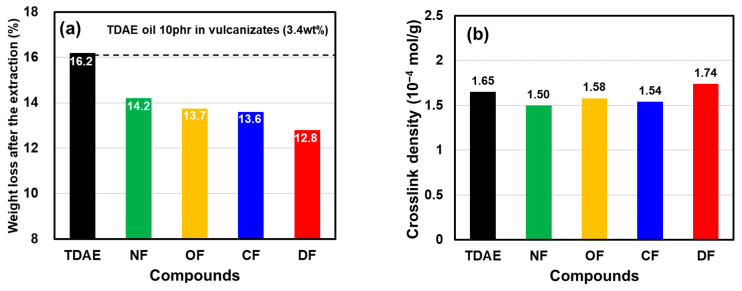
(**a**) Weight loss after extraction and (**b**) crosslink density of the vulcanizates.

Organic matter was extracted from vulcanizate specimens using two types of organic solvents, and the amount of organic matter was determined. First, oils and low-molecular-weight components added during mixing were extracted using THF. Soluble free LqBRs were then extracted using *n*-hexane from the specimen obtained after extraction with THF. The total amount of organic matter extracted using the two different organic solvents is shown in Figure 7a and Table 8.

The proportion of 40 phr of TDAE oils in the vulcanizate specimen was 13.6 wt%, of which 10 phr corresponded to 3.4 wt%. The TDAE compound showed the highest organic matter extraction amount at 16.2 wt% (40 phr of TDAE oil; 13.6 wt% + some additives; 2.6 wt%). This is because the oil can easily be extracted by organic solvents, as it does not form a chemical bond with other materials in the compound. In contrast, approximately 58.8% of NF-LqBR, which has a molecular weight of 25,500 g/mol, can be fixed in the polymer network through co-vulcanization with the base polymers during vulcanization. Therefore, a smaller extraction amount was obtained, as compared with the TDAE compounds. The extraction amount obtained from OF-LqBR and CF-LqBR was smaller than from NF-LqBR because the functional groups can be fixed on the silica surface by the silanization reaction, in addition to co-vulcanization with the base polymer (i.e., LqBR extraction amount: 41.2% versus 26.5% and 23.5%). In particular, in the case of DF-LqBR with 2.2 functional groups per chain, the extraction amount was reduced to 10 phr (3.4 wt%) that replaced TDAE oils (i.e., DF-LqBR extraction amount: 0%). Therefore, it is considered that DF-LqBR was not extracted from the vulcanizate.

The crosslink densities of the vulcanizates are shown in Figure 7b and Table 8. LqBR generally reduces the crosslink density of the vulcanizate because it consumes sulfur for use in the base polymer crosslinking. However, OF-LqBR and CF-LqBR formed chemical bonds between silica and rubber, resulting in higher crosslink density values than that of the NF-LqBR compound. In particular, OF-LqBR has a large entanglement effect because its molecular weight is twice that of the CF-LqBR chain before the coupling reaction [40], which is advantageous for the crosslinking reaction to form a crosslinking point with the base polymer [8]. Therefore, the crosslink density of OF-LqBR was larger than that of CF-LqBR. In the case of DF-LqBR, the network structure formed through self-condensation [5] not only has swelling resistance, but also forms chemical bonds between silica and rubber, even though it has a small molecular weight of 7400 g/mol. Thus, it showed higher crosslink density values, as compared with the OF-LqBR and CF-LqBR compounds.

### 3.7. Mechanical Properties and DIN Abrasion Loss

**Figure 8:**
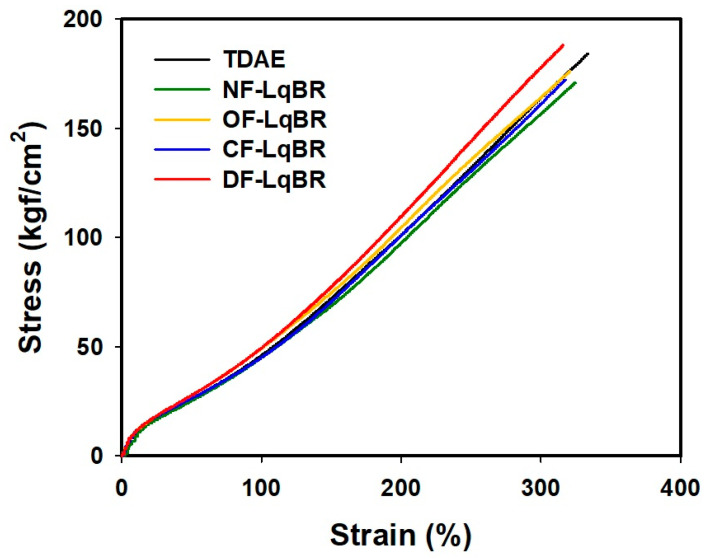
Stress–strain curves of the vulcanizates according to the applied processing aids.

The mechanical properties and DIN abrasion loss measurement results are shown in Figure 8 and Table 9, respectively. It is known that the modulus at 300% elongation (M_300_) in the stress–strain curves is highly correlated with the crosslink density [20]. LqBR generally acts as a lubricant between the base polymer chains to facilitate slippage and decrease the modulus owing to a decrease in the crosslink density value by consuming sulfur. However, the OF-LqBR and CF-LqBR compounds showed similar M_300_ values, as compared with those of the TDAE compound, even though the crosslink density was lower. This is caused not only by increased filler–rubber interactions owing to the coupling reaction of OF-LqBR and CF-LqBR, but also by increased modulus owing to the effect of chain entanglement [19,35]. In the case of DF-LqBR, the crosslink density increased because of the network structure formed by self-condensation, which resulted in the highest M_300_ value.

In general, it is known that abrasion resistance is significantly affected by the T_g_ of the polymer and the filler–rubber interaction [41,42,43,44]. In addition, Seo et al., reported that, in silica-filled compounds, the abrasion resistance according to the macrostructure (linear-shaped SBR versus star-shaped SBR) of the polymer was better with the star-shaped SBR with advantageous silica dispersion [45]. The LqBR, which has lower T_g_ than TDAE oils, showed excellent abrasion resistance in all compounds by lowering the compound T_g_. In addition, F-LqBRs that are capable of the coupling reaction increased the filler–rubber interaction and showed improved abrasion resistance, as compared with the NF-LqBR compounds. The CF-LqBR compound exhibited superior abrasion resistance, as compared with the OF-LqBR compound, as the silica dispersion improved, which was confirmed in the previous Payne effect result. DF-LqBR has excellent silica dispersion and high filler–rubber interaction. However, the degree of improvement in the abrasion resistance was small, as compared to that of OF-LqBR and CF-LqBR owing to the high T_g_ of DF-LqBR. Consequently, the T_g_ of the LqBR, in addition to the silica dispersion improvement and the increase in the filler–rubber interaction, is considered to be the most important factor influencing the abrasion resistance of compounds to which LqBRs are applied.

### 3.8. Dynamic Viscoelastic Properties

To predict the tire performance, the viscoelastic properties of tread compounds can be measured in the laboratory with excellent correlation. Among the viscoelastic properties, the storage modulus (G′) value in the low-temperature region below −10 °C is an indicator of snow traction, and the lower the value, the better the snow traction [46,47]. This is because the tire tread can be deformed and adhered to the icy road surface more easily when the G′ value is lower under low-temperature conditions [48]. The value of the loss modulus (G″) at 0 °C, which is used as an indicator of the tire wet traction, is known to improve the wet traction performance as the value is increased. In addition, the G″ value tends to increase with the effective filler volume fraction [49]. The value of tan δ at 60 °C is an indicator of the rolling resistance of tires, and the lower the value, the better the fuel economy performance [50]. The main energy dissipation in the high-temperature region is due to the destruction and reformation of the filler–filler network [49]. Kitamura et al., reported that free chain ends of the polymers also contribute to hysteresis in this region [51]. Salort et al., reported that when low-molecular-weight butadiene rubber is crosslinked to the base polymers, hysteresis occurs because of dangling chain ends [8]. The results of both studies suggest that free chain ends of the polymers are also an important factor in the energy loss in the high-temperature region.

**Figure 9:**
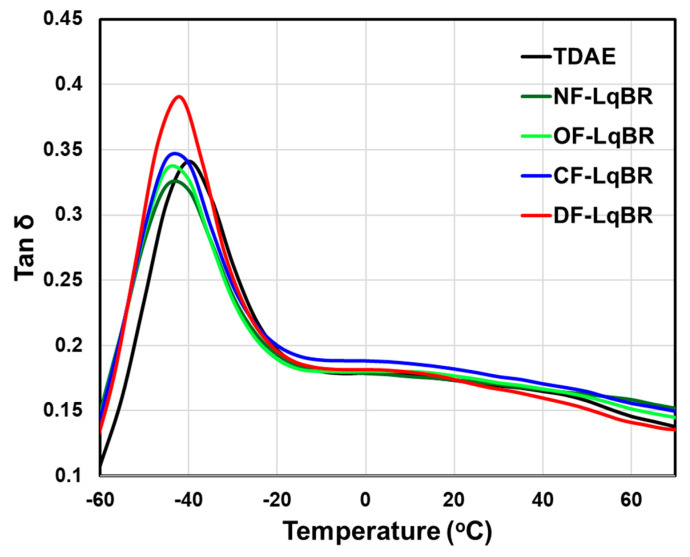
Tan δ curves of the vulcanizates according to the applied processing aids.

Figure 9 and Table 10 show the results of the dynamic viscoelastic properties of the compounds to which TDAE oils or LqBRs are applied. All LqBR compounds showed lower values of G′ at −30 °C than the TDAE compound, regardless of the functional group position. This is because LqBRs with low T_g_ lowered the T_g_ of the compounds and increased the flexibility of the compounds at low temperatures. 

In the case of the value of G″ at 0 °C, all LqBR compounds showed lower values than the TDAE compound, which is inferred to be due to the decrease in the T_g_ of the vulcanizates. In addition, as confirmed from the previous Payne effect results, it is considered that all functionalized LqBRs exhibited lower values of G″ at 0 °C than NF-LqBR because the effective filler volume fraction lowered as the silica dispersion was improved. 

As shown in Table 10, the value of tan δ at 60 °C showed a tendency to decrease as the number of the free chain ends of LqBRs was decreased. NF-LqBR showed a higher value of tan δ at 60 °C than the TDAE compound because the dangling chain ends after vulcanization of NF-LqBR act as free chain ends. CF-LqBR showed a lower value of tan δ at 60 °C than NF-LqBR, as the chain mobility was decreased by the coupling reaction. However, the value was still higher than that of the TDAE compound because of the free chain ends of both ends. In contrast, OF-LqBR and DF-LqBR showed lower values of tan δ at 60 °C than CF-LqBR because the number of free chain ends was decreased, as the chain ends were fixed to the silica surface. In particular, DF-LqBR showed a lower value than the TDAE compound because the occurrence of hysteresis was the least, owing to the absence of free chain ends. From these results, it is concluded that the effect of the free chain ends of LqBRs is dominant in increasing the values of tan δ at 60 °C in the compounds to which LqBR is applied.

### 3.9. The Performance Change of the Compounds

Table 11 shows the tendency of the change in the physical properties of the compound according to the position of the functional group of LqBRs and the number of free chain ends.

## 4. Conclusions

The effects of the functional group position and number of the free chain ends of LqBRs applied as processing aids to silica-filled rubber compounds on the physical properties and hysteresis of the compounds were investigated in this study.

- Payne effect and processability were improved in all functionalized LqBRs without deteriorating the mechanical properties. In particular, CF-LqBR, which has small hydrodynamic volume, showed a lower Payne effect and Mooney viscosity than OF-LqBR. DF-LqBR showed a somewhat high Mooney viscosity owing to the terminal amide and cyano groups.
- NF-LqBR showed a lower delta torque (ΔT) than the TDAE compounds because of the consumption of sulfur during vulcanization, whereas F-LqBRs showed higher delta torque than NF-LqBR because the functional groups not only were fixed on the silica surface, but also formed chemical bonds through a crosslinking reaction with the base polymers.
- The extracted amount of organic matter decreased as the number of functional groups in LqBR was increased.
- The mechanical properties were highly correlated with the crosslink density.
- The T_g_ of LqBR, as well as the increase in the filler–rubber interaction, are considered as major factors affecting abrasion resistance. As for dynamic properties, snow traction improved as LqBRs lowered the T_g_ of the compound. The value of tan δ at 60 °C decreased as the number of the free chain ends of LqBR was decreased.

These characteristics of LqBRs are suitable for winter tires that require high traction and long-term flexibility at low temperatures. In particular, DF-LqBR is a material that can improve fuel efficiency and achieve wear performance simultaneously, and can solve the trade-off problem of tire properties. In brief, DF-LqBR is considered to be an appropriate material for tread compounds of electronic car tires and green tires. In addition, it is expected that the results presented in this study will provide the basis for the optimal LqBR structure design and selection required for the manufacture of tires to which LqBR is applied.

## Figures and Tables

**Table 1:** Formulation for liquid butadiene rubber (LqBR) polymerization.

	NF-LqBR	OF-LqBR	CF-LqBR	DF-LqBR
Butadiene (g)	100	70	70	83.3
Cyclohexane (g)	500	400	400	-
THF (g)	-	-	-	416
n-Butyllithium (mmol)	3.9	2.8	5.7	-
DF initiator (g)	-	-	-	0.529
Anisole (mmol)	11.9	8.4	16.3	-
CPTES (mmol)	-	3.0	-	-
TEOS (mmol)	-	-	2.8	-
n-octyl alcohol (mmol)	4.34	-	-	-

**Table 2:** Formulation of the rubber/silica compounds using processing aids (unit: parts per hundred rubber, phr).

Sample Code	TDAE	NF-LqBR	OF-LqBR	CF-LqBR	DF-LqBR
SSBR	80	80	80	80	80
BR	20	20	20	20	20
Silica	120	120	120	120	120
X50S	20	20	20	20	20
TDAE oil	40	30	30	30	30
LqBRs	0	10	10	10	10
DPG	2	2	2	2	2
ZnO	3	3	3	3	3
Stearic acid	1	1	1	1	1
6PPD	2	2	2	2	2
Sulfur	1.3	1.3	1.3	1.3	1.3
CBS	1.6	1.6	1.6	1.6	1.6

**Table 3:** Mixing procedures.

	Time, min:s	Action
First stage	0:00–0:40	Add rubber (Initial temperature: 100 °C)
	0:40–1:20	Mixing
	1:20–2:20	Add silica 1/2 + X50S 1/2 + DPG 1/2 + oil 1/2 + LqBR 1/2
	2:20–3:20	Mixing
	3:20–4:20	Add silica 1/2 + X50S 1/2 + DPG 1/2 + oil 1/2 + LqBR 1/2
	4:20–5:20	Mixing
	5:20–5:40	Add ZnO, St/A, 6PPD
	5:40–8:00	Mixing
	8:00–9:00	Ram up
	9:00–11:40	Additional mixing and dump (Dump temperature: 150–155 °C)
Second stage	0:00–0:40	Add a master batch from the first stage (Initial temperature: 50 °C)
	0:40–1:00	Mixing
	1:00–1:20	Add curatives
	1:20–3:00	Mixing and dump (Dump temperature: 80–90 °C)

**Table 4:** Characteristics of LqBRs.

	NF-LqBR	OF-LqBR	CF-LqBR	DF-LqBR
Aliquot sample ^a^ M_n_ (g/mol)	-	25,300	13,000	-
Final sample M_n_ (g/mol)	25,500	26,600	24,700	7400
Polydispersity index (PDI)	1.08	1.12	1.14	1.84
Vinyl content (% in BD)	10	8	10	19
T_g_ (°C)	−95	−95	−94	−83
Coupling number (CN)	-	-	1.9	-
Functionality (Si/chain)	-	0.98	1.07	2.22

^a^ Aliquot sample; LqBR sample before addition to TEOS.

**Table 5:** Peak torque of the compounds during the first stage mixing.

Compound	TDAE	NF-LqBR	OF-LqBR	CF-LqBR	DF-LqBR
Peak torque (N-m)	28.1	30.6	29	28.9	25.8

**Table 6:** ΔG′ values of the compounds.

Compound	TDAE	NF-LqBR	OF-LqBR	CF-LqBR	DF-LqBR
ΔG′ (0.28–40.04%, MPa)	6.54	5.47	5.2	5.04	4.23

**Table 7:** Cure characteristics and Mooney viscosity of the compounds.

Compound	TDAE	NF-LqBR	OF-LqBR	CF-LqBR	DF-LqBR
t_10_ (min:s)	01:19	01:01	01:00	01:00	01:14
t_90_ (min:s)	11:00	12:10	11:58	11:38	10:37
T_min_ (N·m)	0.9	0.77	0.75	0.74	0.8
T_max_ (N·m)	3.11	2.73	2.89	2.83	2.92
△T (T_max_–T_min_, N·m)	2.21	1.96	2.14	2.09	2.12
Mooney viscosity (ML_1+4_@100 °C)	154	148	145	141	152

**Table 8:** Weight loss of the vulcanizates after extraction and crosslink density.

Compound.	TDAE	NF-LqBR	OF-LqBR	CF-LqBR	DF-LqBR
Weight loss after the extraction (wt%)	16.2	14.2	13.7	13.6	12.8
Weight loss after the extraction in 10phr of TDAE oil and LqBRs (%)	100	41.2	26.5	23.5	0
Crosslink density (10^−4^ mol/g)	1.65	1.50	1.58	1.54	1.76

**Table 9:** Mechanical properties and DIN abrasion loss of the vulcanizates.

Compound	TDAE	NF-LqBR	OF-LqBR	CF-LqBR	DF-LqBR
M_100_ (kgf/cm^2^)	46	45	49	45	49
M_300_ (kgf/cm^2^)	164	156	164	161	178
Elongation at break (%)	334	325	321	318	316
Tensile strength (kgf/cm^2^)	184	171	176	172	188
DIN abrasion loss (mg)	121	102	99	93	100
Compound T_g_ (°C)	−40.0	−43.2	−43.5	−43.1	−42.3

**Table 10:** Viscoelastic properties of the compounds according to the applied processing aids.

Compound	TDAE	NF-LqBR	OF-LqBR	CF-LqBR	DF-LqBR
T_g_ (°C)	−40.0	−43.2	−43.5	−43.1	−42.3
G′ at −30 °C (MPa)	172	154	155	148	147
G″ at 0 °C (MPa)	12.0	11.5	11.3	11.1	10.7
Tan δ at 60 °C	0.145	0.158	0.151	0.156	0.142
The number of free chain ends	N/A	2	1	2	0

**Table 11:** Effect of the functional group position of F-LqBR on the performance of silica-filled rubber compounds.

Types of Processing Aids	Payne Effect	Extraction Resistance	M_300_	Abrasion Resistance	Tan δ at 60 °C	G′ at −30 °C
TDAE oil (Reference)	M	B	M	M	M	M
NF-LqBR	M	M	B	G	W	G
OF-LqBR	G	G	M	G	B	G
CF-LqBR	G	G	M	E	B	E
DF-LqBR	E	E	E	G	G	E

E: Excellent, G: Good, M: Moderate, B: Bad. W: Worst.

## Data Availability

Data presented in this study are available upon request from the corresponding author.

## Abbreviations

M_c_ (mol/g)	Average molecular weight between crosslink points
ν (mol/g)	Crosslink density
ν_r_	Volume fraction of rubber in the swollen gel at equilibrium
V_s_ (cm^3^/mol)	Molar volume of solvent
ρ (g/cm^3^)	Density of the rubber sample
χ	Polymer–solvent interaction parameter
w_dry_	Weight of the dry sample
w_filler_	Weight of the filler in the dry sample
w_swollen_	Weight of the swollen sample
ρ_rubber_	Density of the rubber
ρ_solvent_	Density of the solvent
ν_0_	Molar volume of the solvent
δ_p_	Solubility parameter of the polymer
δ_s_	Solubility parameter of the solvent
t_10_ (min:s)	Scorch time
t_90_ (min:s)	Optimum cure time
T_min_ (N·m)	Minimum torque
T_max_ (N·m)	Maximum torque
△T (N·m)	Delta torque (T_max_–T_min_)
M_100_ (kgf/cm^2^)	Modulus at 100% elongation
M_300_ (kgf/cm^2^)	Modulus at 300% elongation
T_g_ (°C)	Glass transition temperature
G′ (MPa)	Storage modulus
G″ (MPa)	Loss modulus
Tan δ	Tangent delta
